# Supplementary material for: Inter-Alpha Inhibitor Proteins Modify the Microvasculature after Exposure to Hypoxia–Ischemia and Hypoxia in Neonatal Rats
Source: Int J Mol Sci. 2023 Apr 4;24(7):6743. doi: 10.3390/ijms24076743 (PMC10094872; doi:10.3390/ijms24076743)
Supplement: Supplementary file 1 [file ijms-24-06743-s001.zip › ijms-2264985-supplementary.pdf]

Inter-Alpha Inhibitor Proteins Modify the Microvasculature after Exposure to Hypoxia–Ischemia and Hypoxia in Neonatal Rats

Francesco Girolamo [1], Yow-Pin Lim [2,3], Daniela Virgintino [1], Barbara S. Stonestreet [4] and Xiaodi F. Chen [4,*]

[1] Department of Translational Biomedicines and Neuroscience (DiBraiN), University of Bari School of Medicine, 70124 Bari, Italy
[2] ProThera Biologics, Inc., Providence, RI 02905, USA
[3] Department of Pathology and Laboratory Medicine, Alpert Medical School of Brown University, Providence, RI 02905, USA
[4] Women & Infants Hospital of Rhode Island, Alpert Medical School of Brown University, Providence, RI 02905, USA
* Correspondence: xchen@wihri.org; Tel.: +1-(401)-274-1122 (ext. 48025)

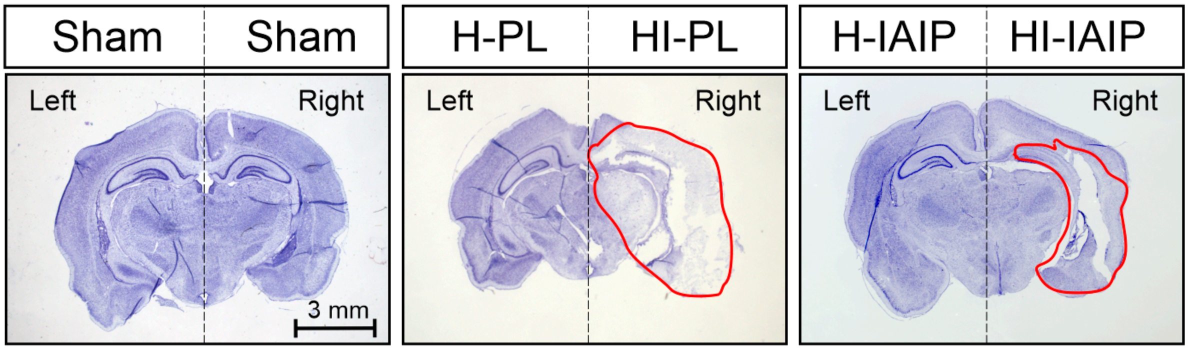

Supplementary Figure S1:

Representative cresyl violet images of the coronal brain sections from the Sham, H/HI-PL, and H/HI-IAIP stained with cresyl violet 3 days after HI brain injury. Infarct areas were contoured with the red line. Scale bar = 3 mm. This Nissl-stained image is distinct from previous published Nissl images [1], which were obtained from neonatal rats using the same hypoxic-ischemic and hIAIP treatment procedures as in the current study.

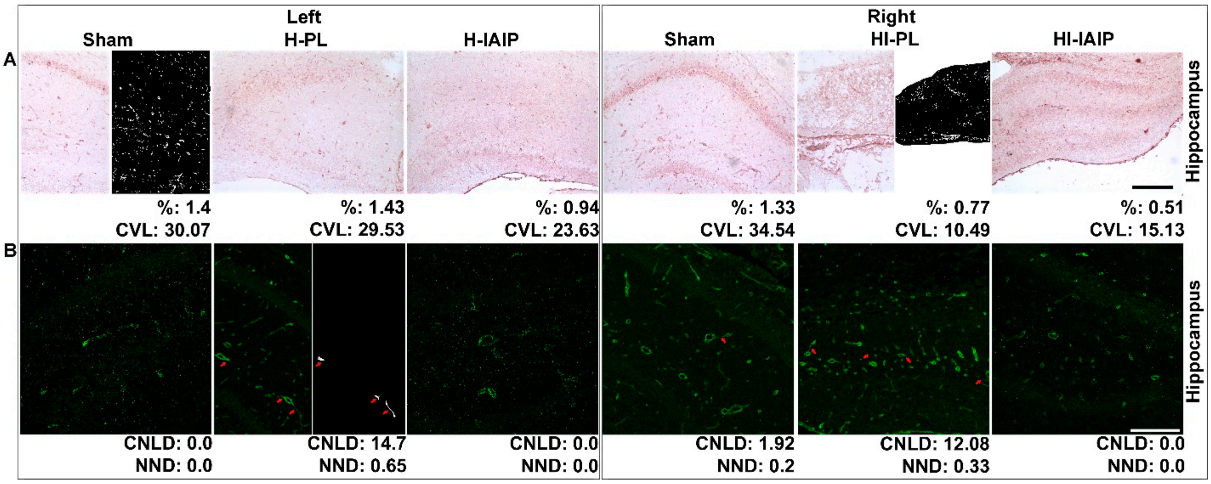

Supplementary Figure S2:

Representative images used in the quantitative analyses comparing sham-operated, hypoxia (H, left side of brain) and hypoxia-ischemia (HI, right side of brain) in a male neonatal rat brain vasculature.

A) Representative laminin immunohistochemical labeling and thresholded binary images of coronal hippocampal sections (acquired with a 10x lens light microscope) used to automatically measure the percent (%) of the laminin-stained area and the cumulative laminin$^+$ vessel length (CVL: expressed as multiples of 100 μm of vessel length)/area (1 mm^2). Scale bar: 250 μm; field total area 975,440 μm^2.

B) Representative immunofluorescence confocal laminin immunolabeling and thresholded binary images of coronal hippocampal sections (acquired with a 40x lens confocal microscope) used to localize (red arrows) and automatically measure the cumulative laminin$^+$ nanotube linear density (CNLD: expressed in μm/acquired volume expressed as 10^6 μm^3) and the nanotube numerical density (NND: number of identified nanotubes/acquired volume). Scale bar: 100 μm; field total volume 1.201 10^6 μm^3.

References:

1. Chen, X.; Nakada, S.; Donahue, J. E.; Chen, R. H.; Tucker, R.; Qiu, J.; Lim, Y. P.; Stopa, E. G.; Stonestreet, B. S., Neuroprotective effects of inter-alpha inhibitor proteins after hypoxic-ischemic brain injury in neonatal rats. *Exp Neurol* **2019,** 317, 244-259.
